# Anticancer Activity of Sunitinib Analogues in Human Pancreatic Cancer Cell Cultures under Normoxia and Hypoxia

**DOI:** 10.3390/ijms24065422

**Published:** 2023-03-12

**Authors:** Ieva Skaraitė, Elias Maccioni, Vilma Petrikaitė

**Affiliations:** 1 Laboratory of Drug Targets Histopathology, Institute of Cardiology, Lithuanian University of Health Sciences, Sukileliu pr. 13, LT-50162 Kaunas, Lithuania; 2 Department of Life and Environmental Sciences, University of Cagliari, Via Ospedale 72, 09124 Cagliari, Italy

**Keywords:** pancreatic cancer, cell viability, cell migration, hypoxia, cell colony formation, sunitinib, isatin-dihydropyrazole

## Abstract

Pancreatic cancer remains one of the deadliest cancer types. It is usually characterized by high resistance to chemotherapy. However, cancer-targeted drugs, such as sunitinib, recently have shown beneficial effects in pancreatic in vitro and in vivo models. Therefore, we chose to study a series of sunitinib derivatives developed by us, that were proven to be promising compounds for cancer treatment. The aim of our research was to evaluate the anticancer activity of sunitinib derivatives in human pancreatic cancer cell lines MIA PaCa-2 and PANC-1 under normoxia and hypoxia. The effect on cell viability was determined by the MTT assay. The compound effect on cell colony formation and growth was established by clonogenic assay and the activity on cell migration was estimated using a ‘wound healing’ assay. Six out of 17 tested compounds at 1 µM after 72 h of incubation reduced cell viability by 90% and were more active than sunitinib. Compounds for more detailed experiments were chosen based on their activity and selectivity towards cancer cells compared to fibroblasts. The most promising compound EMAC4001 was 24 and 35 times more active than sunitinib against MIA PaCa-2 cells, and 36 to 47 times more active against the PANC-1 cell line in normoxia and hypoxia. It also inhibited MIA PaCa-2 and PANC-1 cell colony formation. Four tested compounds inhibited MIA PaCa-2 and PANC-1 cell migration under hypoxia, but none was more active than sunitinib. In conclusion, sunitinib derivatives possess anticancer activity in human pancreatic adenocarcinoma MIA PaCa-2 and PANC-1 cell lines, and they are promising for further research.

## 1. Introduction

Cancer is one of the leading causes of death worldwide. According to the International Agency for Research on Cancer, in 2020 there were over 19 million new cases worldwide and almost 10 million deaths related to oncological diseases [1]. Adenocarcinoma accounts for more than 90% of all pancreatic malignancies [2].

The exceptionally poor prognosis of pancreatic cancer is mainly associated with a late diagnosis because of non-specific symptoms, rapid tumour progression, early and aggressive metastasis, and high resistance to chemotherapy [3,4]. The most reliable treatment for pancreatic adenocarcinoma is surgical removal, but more than 80% of patients are diagnosed with metastatic pancreatic adenocarcinoma. Therefore, surgical removal is not always possible. In this case, the average survival is from 12 to 18 months [5]. In patients diagnosed with a resectable tumour, the prognosis remains poor, and five-year survival is only 20% [6]. Overall, the five-year survival rate for pancreatic adenocarcinoma is just 9% [7]. Nevertheless, antiangiogenic treatment is still considered promising [8].

Most pancreatic ductal adenocarcinomas have been found to be caused by primary cystic lesions of the pancreas called neoplasms. Pancreatic intraductal papillary mucin neoplasms and pancreatic intraepithelial neoplasms gradually progress to the acquisition of genetic changes and culminate in the onset of pancreatic adenocarcinoma [9]. Intraepithelial neoplasms have mutations in the Kirsten mouse sarcoma virus oncogene homologue (KRAS) detected in approximately 90% of patients with pancreatic adenocarcinomas. Mutation of this oncogene activates the intercellular signalling pathways of RAS proteins and phosphatidylinositol 3-kinase (PI3K), which regulate the cell cycle, leading to increased tumour growth, cancer cell survival, and motility [10].

Increased expression of vascular endothelial growth factor (VEGF), vascular endothelial growth factor receptor (VEGFR) and platelet-derived growth factor β-receptor (PDGFRβ) have been observed in pancreatic adenocarcinoma cells. This results in increased tumour angiogenesis and increases the incidence of metastases [11,12]. Increased PDGFR expression promotes the proliferation of pericytes and fibroblasts, leading to tumour progression [13]. VEGF is a potent factor in the proliferation and angiogenesis of vascular endothelial cells, promoting the vascularization required for primary tumour growth and metastasis [14]. Thus, increased VEGF synthesis is associated with low oncological survival and recurrence [13].

Studies have shown that multitargeted tyrosine-kinase inhibitor (MTKI) sunitinib inhibits tumour vascularization and cell viability [15]. Sunitinib malate is a small molecule that acts as a kinase inhibitor [16]. It is a potent type IV: VEGFR1, VEGFR2, VEGFR3 and type III: PDGFRα, PDGFRβ, stem cell factor (c-KIT), Fms-like tyrosine kinase 3 (FLT3) and colony-stimulating factor receptor (CSF-1R), receptor tyrosine kinase (RTK) inhibitor [17,18]. Sunitinib has a plane aromatic ring mimicking the adenine group of ATP, which binds strongly to the ATP-binding gap, disrupting kinase function [16]. For example, the binding of VEGF to VEGFR results in the dimerization of VEGFR. This results in the activation of the intracellular VEGFR kinase domain, but ATP is required to activate the further signalling path. The action of sunitinib is competitive; it competes with ATP for VEGFR ATP-binding ‘pocket’. Therefore, activated VEGFR can no longer activate the intracellular kinase domain [16].

Thus, sunitinib has multiple inhibitory effects on RTK. Inhibition of RTK inhibits signal transduction, thereby inhibiting PI3K, RAS, and protein kinase C (PKC) signalling pathways. Thus, tumour growth, progression, angiogenesis, and metastasis are inhibited [19].

Tissue hypoxia is common in solid tumours with high oxygen demand and low vascularization [20]. In the environment of pancreatic cancer, the mean partial oxygen pressure (pO_2_) was found to be 0–5.3 mmHg (0–0.7%), which is a significantly lower amount of oxygen compared to normal pancreatic tissue with a pO_2_ of 24.3–92.7 mmHg (3.2–12.3%) [21]. The microenvironment of the pancreatic adenocarcinoma is characterized by a stroma composed of densely packed fibroblasts and inflammatory cells, resulting in hypoxia due to limited oxygen uptake [22]. Oxygen uptake is increased by the rapid proliferation of cancer cells, and lack of vascularization reduces oxygen supply [23]. Pancreatic adenocarcinoma cells adapt to hypoxic conditions by activating transcription factors, such as hypoxia-inducible factor (HIF). The latter, in turn, stimulates the expression of related genes involved in angiogenesis and glycolysis [22].

Tyrosine kinase receptors, including fibroblast growth factor (FGF), PDGF, VEGF, and their ligand receptors, are known to play an essential role in tumour growth and angiogenesis. Thus, exposure to RTK with certain receptor antagonists or antibodies may cause anticancer effects [16]. Studies have shown that hypoxic cells are more resistant to both chemotherapy and radiotherapy and may increase the potential for metastasis [24,25]. Therefore, it is important to evaluate compound effects under hypoxic conditions.

For the experiments were selected two pancreatic adenocarcinoma cell lines MIA PaCa-2 and PANC-1. These are the two most used tumour cell lines in pancreatic adenocarcinoma studies in vitro [26]. Both lines are derived from primary pancreatic adenocarcinoma [27]. The doubling time of MIA PaCa-2 and PANC-1 cells is 40 and 52–56 h, respectively [28,29]. Both cell lines contain mutations in the KRAS oncogene, resulting in resistance to chemotherapy [30]. The MIA PaCa-2 cell line has higher concentrations of alanine, glycine, and leucine compared to the PANC-1 cell line, indicating a greater need for protein synthesis [31]. MIA PaCa-2 cells also die by a caspase-dependent apoptotic pathway, contrary to PANC-1 cells which die by a caspase-independent apoptotic mechanism [32]. The PANC-1 cell line is characterized by higher expression of electron transport chain proteins in mitochondria resulting in the higher respiratory activity of PANC-1 cells compared to MIA PaCa-2 [33]. 

Both MIA PaCa-2 and PANC-1 cells express the epithelial markers CK5.6 and AE1/AE3, and the mesenchymal marker vimentin, allowing them to be described as epithelial-mesenchymal cells. PANC-1 has been shown to lack E-cadherin protein expression [26]. Increased expression of E-cadherin is associated with better survival in oncological disease, and the PANC-1 cell line is considered to be more aggressive and has a higher metastatic potential [34]. Cytometric analysis and immunocytochemistry have also shown that the PANC-1 cell line secretes more stem cell surface markers. Therefore, the PANC-1 cell line has a higher ability to regenerate compared to the MIA PaCa-2 cell line [34].

**Figure 1:**
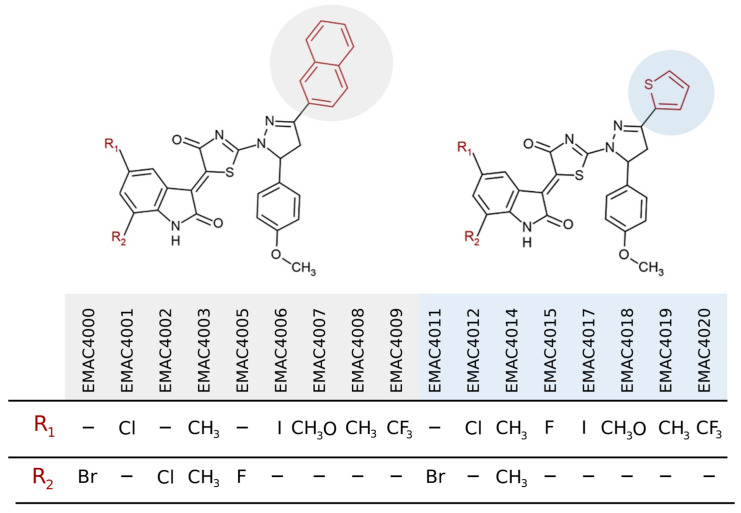
Structural formulas of sunitinib derivatives.

The chosen sunitinib derivatives were already published in our previous research (Figure 1) [35]. This study aimed specifically to assess the potential of a series of isatin-dihydropyrazole hybrids as anticancer agents in pancreatic cancer studying them in different assays (clonogenic assay, ‘wound’ healing assay). 

## 2. Results and Discussion

### 2.1. Sunitinib Analogues Reduce the Viability of Pancreatic Cancer Cells under Hypoxia and Normoxia Conditions

**Figure 2:**
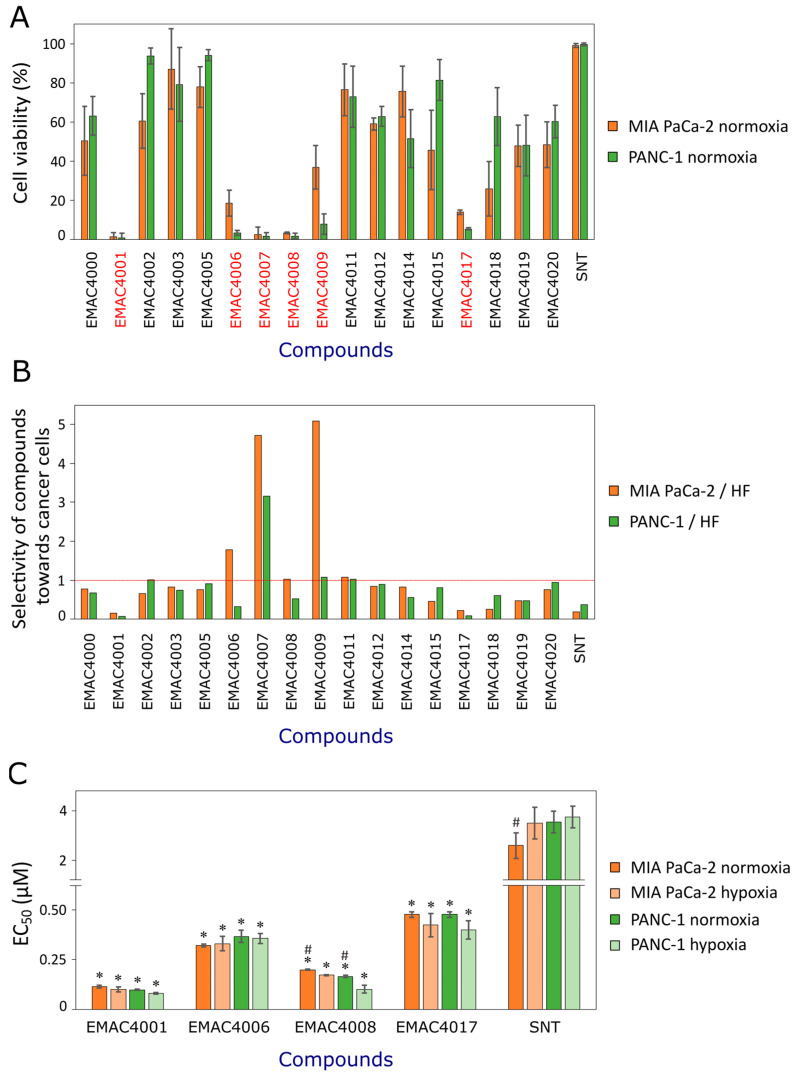
Effects of compounds on the viability of MIA PaCa-2 and PANC-1 cells. (**A**). Effect of sunitinib and its derivatives at 1 µM solutions on the viability of pancreatic cancer MIA PaCa-2 and PANC-1 cells. The most active compounds are indicated in red color. (**B**). Selectivity of compounds towards cancer cells compared to human fibroblasts. (**C**). Comparison of EC_50_ values of compounds under normoxia and hypoxia. Asterisks (*) indicate *p* < 0.05 compared to the control, (#) *p* < 0.05 compared between the same compound activity in normoxia and hypoxia. Abbreviations: SNT—sunitinib, HF—human fibroblasts.

The effect on cell viability was determined by the MTT assay. Compounds EMAC4001, 4006, 4007, 4008, 4009 and 40017 at 1 µM concentration after 72 h of incubation reduced the viability of at least one cell line by 90% and statistically significantly stronger reduced the viability of both cancer cell lines compared to sunitinib (Figure 2A). Also, the selectivity of sunitinib derivatives against cancerous compared to non-cancerous cells (human fibroblasts) was established (Figure 2B). Not all compounds inhibited cancer cells more strongly compared to human fibroblasts. Compounds EMAC4007 and 4009 were found to significantly reduce the viability of non-cancerous cells. Half-maximal effective concentration (EC_50_) values were calculated for the most active and selective compounds EMAC4001, 4006, 4008, 40017 in normoxia and hypoxia (Figure 2C). All of them had different substituents at the 5-position of isatin and they also were identified as the most active ones of another pancreatic cell line BxPC-3 in our previous study [35].

All tested compounds inhibited cell viability statistically significantly compared to sunitinib (Figure 2C). The most active one was compound EMAC4001. Its EC_50_ values in MIA PaCa-2 and PANC-1 cell lines were 113 ± 8 and 98 ± 3 nM (in normoxia) and 100 ± 13 and 80 ± 5 nM (in hypoxia). EMAC4008 showed statistically significant differences in its effects on MIA PaCa-2 and PANC-1 under normoxia and hypoxia conditions. Sunitinib possessed the lowest antiproliferative activity (EC_50_ values in MIA PaCa-2 and PANC-1 cell lines were 2.67 ± 0.21 and 3.53 ± 0.21 μM in normoxia, and 3.50 ± 0.30 and 3.73 ± 0.21 μM in hypoxia, respectively). 

No statistically significant difference was observed when comparing the effects of compounds between cell lines. Compound EMAC4006 and sunitinib inhibited more efficiently the cell viability of the MIA PaCa-2 cell line while compounds EMAC4001 and 4008 were more active in the PANC-1 cell line. PANC-1 cells are characterized by higher aggressiveness, therefore the greater inhibitory effect of the test compounds against this line is very important. Also, compound EMAC4008 was more active against both cell lines under hypoxia conditions. In contrast, sunitinib was more active under normoxia than hypoxia conditions. Another tyrosine kinase inhibitor imatinib showed the highest activity in the MIA PaCa-2 cell line (EC_50_ value was 9 μM), while the other pancreatic cancer cell line AsPC-1 cell line was the most resistant to imatinib (EC_50_ value was 20 μM) [36]. Gefitinib at 10 μM reduced the viability of PANC-1 cells by 78% [37]. Meanwhile, an almost three times lower concentration of sunitinib (3.5 μM) decreased the PANC-1 cell viability up to 50% and was more active than other described tyrosine kinase inhibitors.

For further research, we chose to use the compound concentrations equal to 10% and 90% from their calculated EC_50_ values, to avoid the possible misinterpretation of other effects in clonogenic and migration assays due to different possible effects on cell viability.

### 2.2. Sunitinib Analogues Suppress Pancreatic Cancer Cell Colony Formation under Hypoxia and Normoxia

**Figure 3:**
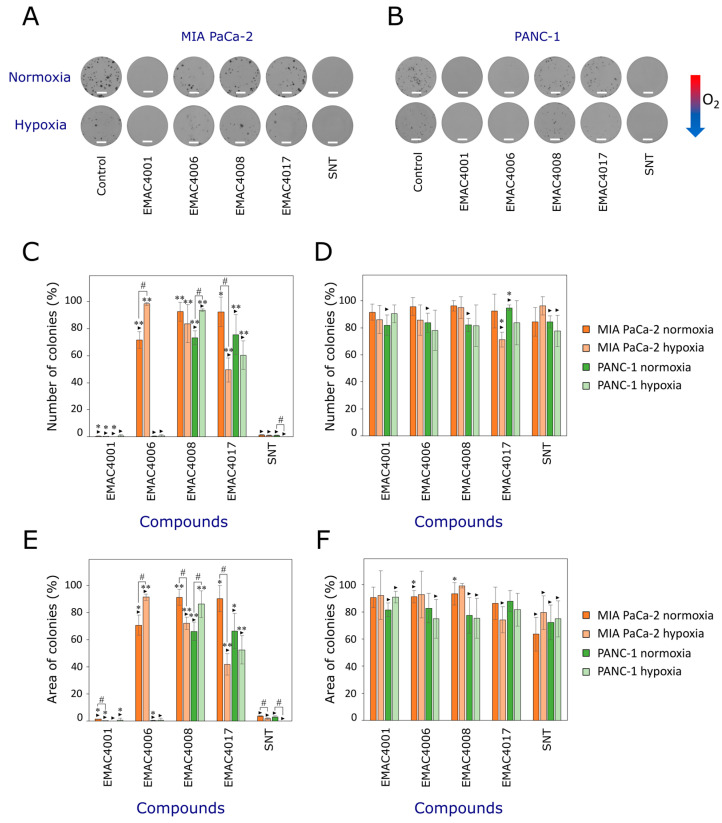
Effects of sunitinib and its derivatives on MIA PaCa-2 and PANC-1 cell colony formation. (**A**). Colonies of MIA PaCa-2 and (**B**). PANC-1 cells after incubation with 90% of EC_50_ under normoxia and hypoxia. (**C**). Effect of compounds at 90% of EC_50_ on the number of MIA PaCa-2 and PANC-1 cell colonies. (**D**). Effect of compounds at 10% of EC_50_ on the number of MIA PaCa-2 and PANC-1 cell colonies. (**E**). Effect of compounds at 90% of EC_50_ on the area of MIA PaCa-2 and PANC-1 cell colonies. (**F**). Effect of compounds at 10% of EC_50_ on the area of MIA PaCa-2 and PANC-1 cell colonies. The scale bar is equal to 5 mm. Asterisks (‣) indicates *p* < 0.05 compared to the control; (*) indicates *p* < 0.05 and (**) *p* < 0.001 compared to sunitinib; (#) indicates *p* < 0.05 compared to the effects of the same compound in normoxia and hypoxia. Abbreviations: SNT—sunitinib.

MIA PaCa-2 formed larger colonies compared to the PANC-1 cell line. Also, MIA PaCa-2 and PANC-1 cell colonies were smaller under hypoxia than under normoxia conditions (Figure 3A,B). 

In the clonogenic assay, the most active compound was EMAC4001. There were less of MIA PaCa-2 and PANC-1 cell colonies and they were smaller in the presence of 90% of EC_50_ of EMAC4001 under normoxia and hypoxia conditions compared to sunitinib.

All tested compounds at 90% of their EC_50_ reduced the number of PANC-1 cell colonies compared to the control under normoxia (Figure 3C,E). Compounds EMAC4001 and 4006 were the most active ones in this assay. Huguet et al. aimed to evaluate the effect of another tyrosine kinase inhibitor afatinib on pancreatic adenocarcinoma cell colony formation. Afatinib did not show an effect at concentrations from 0.25 to 2 μM in PANC-1 cells, but it inhibited cell colony formation of BxPC-3 and Capan-2 cell lines in a concentration-dependent manner [38]. Meanwhile, in our study, compounds EMAC4001 and 4006 inhibited the colony formation of PANC-1 cells even at 5–30 times lower concentrations. 

In contrast to PANC-1 cells, MIA PaCa-2 cell colony formation under normoxia conditions was statistically significantly inhibited only by compounds EMAC4001, 4006, and sunitinib at 90% of their EC_50_ values (Figure 3C,E). Bartscht et al. evaluated the effect of another tyrosine kinase inhibitor, dasatinib, on the PANC-1 cell colony formation. Dasatinib at 10 μM significantly reduced the number of PANC-1 cell colonies [39]. After treatment with gemcitabine in MIA PaCa-2 and PANC-1 cell lines, gemcitabine at 5 μM statistically significantly reduced the colony formation of both cell lines [40]. The methods used by other researchers to study the effects of compounds on cell colony formation were different, thus the results cannot be directly compared. However, in other studies, compounds inhibited colony formation statistically significantly at higher concentrations than those sunitinib analogues used in our study.

In general, under hypoxia, sunitinib more efficiently inhibited the colony formation of both cell lines than under normoxia. Under hypoxia, most of the compounds did not show a statistically significant effect on PANC-1 cell colony formation, in contrast to the MIA PaCa-2 cell line. Compounds EMAC4001, 4008, and 4017 had a stronger effect on the MIA PaCa-2 cell colony growth under hypoxia than normoxia (Figure 3D,F). Under hypoxia, compound EMAC4017 significantly reduced the MIA PaCa-2 cell colony formation. A lower concentration of EMAC4017 reduced the MIA PaCa-2 colony number under hypoxia stronger than sunitinib. Other compounds at 10% of EC_50_ value, were less active than sunitinib and did not reduce the number and area of cell colonies more efficiently. 

### 2.3. Sunitinib Analogues Reduce the Migration of Pancreatic Cancer Cells under Hypoxia and Normoxia 

**Figure 4:**
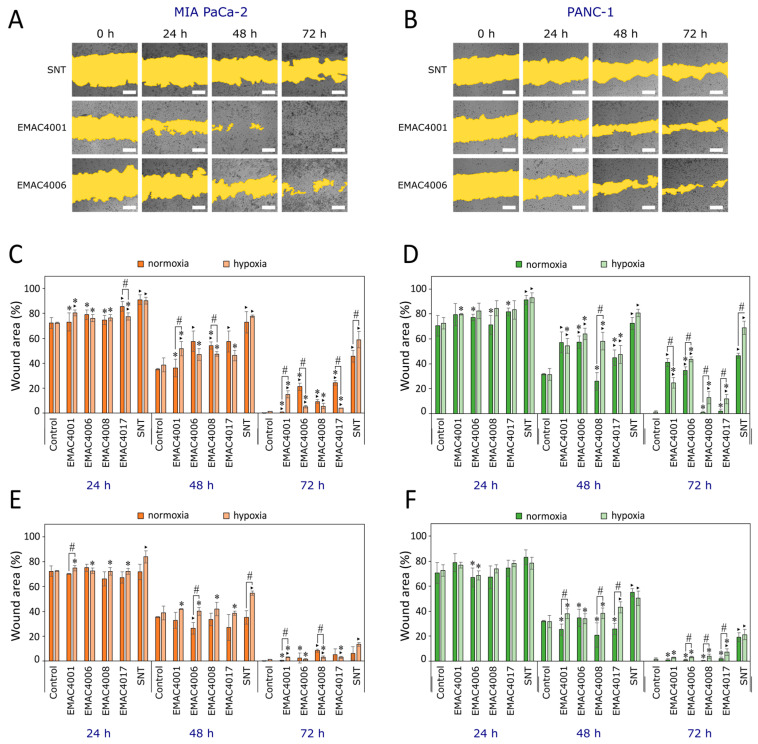
Effects of sunitinib and its derivatives on MIA PaCa-2 and PANC-1 cell migration. (**A**). ‘Wound’ area in MIA PaCa-2 cells at 90% of EC_50_. (**B**). ‘Wound’ area in PANC-1 cells at 90% of EC_50_. (**C**). Effects of compounds on MIA PaCa-2 cell migration at 90% of EC_50_. (**D**). Effect of compounds on PANC-1 cell migration at 90% of EC_50_. (**E**). Effect of compounds on MIA PaCa-2 cell migration at 10% of EC_50_. (**F**). Effect of compounds on PANC-1 cell migration at 10% of EC_50_. Scale bar indicates 100 μm. Asterisks: (‣) indicates *p* < 0.05 compared to the control; (*) indicates *p* < 0.05 compared to sunitinib; (#) indicates *p* < 0.05 compared to effects of the same compound in normoxia and hypoxia. Abbreviations: SNT—sunitinib.

The effect of compounds on cell migration was evaluated by the ‘wound healing’ assay, comparing the changes in the ‘wound’ area (Figure 4A,B). It was found that sunitinib at 10 and 90% of its EC_50_ (EC_50_ values in MIA PaCa-2 and PANC-1 cell lines were 2.67 ± 0.21 and 3.53 ± 0.21 μM in normoxia, and 3.50 ± 0.30 and 3.73 ± 0.21 μM in hypoxia, respectively) (except for the effect of sunitinib at a lower concentration on the MIA PaCa-2 line under normoxia), statistically significantly inhibited cell migration of both lines under normoxia and hypoxia conditions. After 72 h, the ‘wound’ areas in MIA PaCa-2 and PANC-1 cell monolayers grown under hypoxia conditions were 58.90 ± 6.61 and 68.81 ± 5.24%, respectively (Figure 4C–F).

Other researchers observed the change in the ‘wound’ area after 24 h in PANC-1 cell monolayer treated with 1 μM sunitinib, but did not establish a significant difference compared to the control [41]. Gemcitabine at 1 μM concentration did not show a significant difference in ‘wounds’ made in MIA PaCa-2 and PANC-1 cell lines after 24 h and 48 h of incubation compared to the control, as well [42]. However, in our study, there was no difference in ‘wound’ areas in PANC-1 cells incubated with 0.35 μM of sunitinib after 24 h compared to the control, but after longer incubation (48 h and 72 h) the area of the ‘wound’ was significantly larger in the presence of sunitinib (Figure 4D,F). We could hypothesize that the results obtained in different studies could be affected by a different mechanism of action of the compounds, and the long exposure of the drugs could be important.

All tested compounds at 90% of their EC_50_ statistically significantly inhibited MIA PaCa-2 cell migration compared to the control after 72 h (Figure 4C), but this effect was lower than that of sunitinib. Under hypoxia, all compounds at higher concentrations inhibited cell migration stronger compared to the control. Under normoxia, only compounds EMAC4001 and 4006 inhibited cell migration at higher concentrations. All compounds inhibited cell migration at lower and higher concentrations after 72 h, but less efficiently than sunitinib, except the compound higher concentration of EMAC4001 under normoxia. 

Under hypoxia, the effect of compounds EMCA4001, 4006, and 4017 at higher tested concentrations was different. Compounds EMAC4006 and 4017 inhibited the migration of MIA PaCa-2 cells under hypoxia less than under normoxia. Meanwhile, the compounds EMAC4006, 4017, and 4008 reduced the migration of PANC-1 cells stronger under hypoxia than normoxia. In hypoxia, EMAC4001 inhibited the migration of MIA PaCa-2 more than in normoxia, but the effect was the opposite in PANC-1 cells. Sunitinib inhibited the cell migration in both cell lines more under hypoxic conditions.

Based on the obtained results, it can be stated that of the tested compounds, compound EMAC4006 had the greatest effect on inhibiting the migration of both cell lines by a ‘wound healing’ assay. This compound had a greater effect on the migration of the PANC-1 cell line. Compounds inhibited PANC-1 cells more under hypoxia, and MIA PaCa-2 cells under normoxia conditions.

## 3. Materials and Methods

### 3.1. Chemical Materials

EMAC compounds were synthesized by the Maccioni E. group (University of Cagliari, Italy) and published earlier [36]. Sunitinib malate was purchased from Sigma-Aldrich Co. (St Louis, MO, USA).

### 3.2. Cell Culture

The human pancreatic adenocarcinoma MIA PaCa-2 and PANC-1 cell lines were obtained from the American Type Culture. Collection (ATCC, Manassas, VA, USA). MIA PaCa-2 and PANC-1 were cultured in Dulbecco’s Modified Eagle’s GlutaMAX medium (Gibco, Carlsbad, CA, USA). Medium was supplemented with 10% foetal bovine serum (Gibco) 1% antibiotic solution (10,000 units/mL of penicillin and 10 mg/mL of streptomycin, Gibco). Cells were incubated at 37 °C in a humidified atmosphere containing 5% CO_2_.

### 3.3. Cell Viability

Cell viability was tested using MTT (3-(4,5-dimethylthiazol-2-yl)-2,5-diphenyltetrazolium bromide; Sigma-Aldrich Co.) assay, as described previously [43] Briefly, MIA PaCa-2 and PANC-1 cells were seeded in 96-well plates (Corning, NY, USA) in a volume of 100 μL (4000 cells/well). After 24 h incubation, the cells were treated with sunitinib and its derivatives. Only the medium without cells was used as a positive control, and the medium with 0.5% DMSO (Sigma-Aldrich Co.) served as a negative control. After 72 h the cells were incubated with the 0.5 mg/mL of MTT solution for the next 4 h. The coloured formazan product was dissolved in 100 μL DMSO. The absorbance was measured spectrophotometrically at wavelengths 570 and 630 nm. EC_50_ values that represent the concentration of a compound causing a 50% reduction of cancer cell metabolic activity have been calculated using the Hill equation. When determining the EC_50_ values of the compounds under hypoxic conditions, the growth medium was supplemented with 100 µM of CoCl_2_ solution (97%, Alfa Aesar, MA, USA).

### 3.4. Cell Migration

Cell migration was tested by the ‘wound’ healing assay, as described elsewhere [44]. Briefly, MIA PaCa-2 and PANC-1 cells were seeded into 24-well plates (Corning). MIA PaCa-2 at a density of 80,000 cells/well and PANC-1 at a density of 60,000 cells/well in 500 μL of medium and incubated in a humidified atmosphere containing 5% CO_2_ at 37 °C for 1 day. Then the monolayers were scratched with a sterile 100 μL pipette plastic tip in the centre of the well. The old cell medium is aspirated, the wells are rinsed with 400 μL phosphate-buffered saline (PBS, Gibco). Then fresh medium containing compound concentrations representing 10% and 90% of the calculated EC_50_ value was added. Photos of ‘wounds’ were taken every 24 h for 3 days and the compound effect was evaluated by measuring the size of the “wound” area. Images were analysed by TScratch software (CSE Lab, Zurich, Switzerland).

### 3.5. Cell Colony Formation

Compound effect on cell colony formation and growth was established by clonogenic assay, as previously published [45]. Briefly, MIA PaCa-2 and PANC-1 cells were seeded into 12-well plates (Corning) at a density of 200 cells/well in 1 mL of medium and treated with sunitinib derivatives at the concentrations representing 10% and 90% of calculated EC_50_ value. Cells were incubated in a humidified atmosphere containing 5% CO_2_ at 37 °C and grown for 8 days. After incubation, the cells were rinsed with PBS and fixed with 10% formaldehyde, methanol-free (ThermoFisher Scientific, Waltham, MA, USA) solution for 20 min. Then the cells were rinsed with PBS two more times, and cell colonies were stained with 0.1% aqueous crystal violet solution (Sigma-Aldrich Co.) for 20 min, followed by rinses with sterile deionized water to remove the excess dye. Photos of colonies were taken using the G:BOX gel documentation system (Syngene International Ltd., Bengaluru, India) and Genesys software (Syngene International Ltd.). The number of colonies and their area were calculated using GeneTools colony and cell counting software (Syngene International Ltd.) according to the manufacturer’s instructions. Cell colony area and number in control groups were normalized to 100%. The percentage of drug-treated colony area and number were compared to the control.

### 3.6. Statistical Analysis

All experiments were repeated at least three times, calculating the mean and standard deviation. The data were processed using Microsoft Office Excel 2019 software (Microsoft Corporation, Redmond, WA, USA). The level of statistical significance was set at *p* < 0.05.

## 4. Conclusions

In summary, it can be stated that the effects of the compounds in normoxia and hypoxia conditions in MIA PaCa-2 and PANC-1 cell lines are different. EMAC4001 possessed the strongest antiproliferative effect in tested pancreatic adenocarcinoma cell lines and was the most selective compound in MIA PaCa-2 and PANC-1 cell lines. 

All sunitinib derivatives at higher concentrations (except for compounds EMAC4008 and 4017 in the MIA PaCa-2 line), under normoxia conditions, showed an MIA PaCa-2 and PANC-1 cell colony formation inhibiting effect. EMAC4001 and sunitinib at a higher concentration significantly reduced the number and area of colonies in both cell lines under normoxia and hypoxia. Therefore, they could be suitable for the therapy of more aggressive, surviving cells and work in the presence of tissue hypoxia. 

Hypoxia is the main biological factor that causes tumour resistance to chemotherapy, therefore the stronger effect of compounds in hypoxia is very important. When comparing the effects of the compounds with sunitinib, none of the sunitinib derivatives inhibited cell migration more than the parent compound. Therefore, it could be hypothesized that sunitinib analogues would be more suitable for primary, non-metastatic pancreatic cancer treatment.

## Data Availability

The datasets used and/or analysed during the current study are available from the corresponding author on reasonable request.
